# Substituting Resistance Spot Welding with Flexible Laser Spot Welding to Join Ultra-Thin Foil of Inconel 718 to Thick 410 Steel

**DOI:** 10.3390/ma15093405

**Published:** 2022-05-09

**Authors:** Nikhil Kumar, Sisir Dhara, Iain Masters, Abhishek Das

**Affiliations:** 1 Warwick Manufacturing Group, The University of Warwick, Coventry CV4 7AL, UK; nikhil.kumar@warwick.ac.uk (N.K.); sisir.dhara@warwick.ac.uk (S.D.); i.g.masters@warwick.ac.uk (I.M.); 2 Mechanical Engineering Department, Indian Institute of Technology Delhi, New Delhi 110016, India

**Keywords:** micro-resistance spot welding, micro-laser spot welding, ultra-thin foil, joint strength, fusion zone, high-temperature performance

## Abstract

This paper investigated various aspects of replacing existing micro-resistance spot welding (micro-RSW) with micro-laser spot welding for joining Inconel 718 thin foils to thick 410 steel stack-up to allow faster, non-contact joining together with flexibility in spot positioning and removal of tip dressing required for RSW electrodes. The joint quality was evaluated based on the mechanical strength, microstructural characteristics and joint strength at elevated temperature as these joints are often used for high-temperature applications. Experimental investigations were performed using micro-RSW and micro-laser spot welding to obtain the 90° peel and lap shear specimens, each comprising four spots. The obtained strength from laser joints was significantly higher than that of micro-RSW joints due to larger weld nugget formation and interface width. The process map for obtaining good quality welds was also identified, and about a 17% reduction in joint strength was obtained when welded specimens were subjected to elevated temperature (i.e., 500 °C) in comparison with room temperature. This reduction was compensated for using the flexibility of laser welding to add two extra spots. The overall performance of the micro-laser spot welds was found to be better than the micro-RSW considering joint strength, flexibility in placing the spots and time to produce the welds.

## 1. Introduction

Many industrial applications use thin foils to cover parts or components which are exposed to elevated temperature or corrosive environments. This helps to protect the parts/components and increases the functional performance with a substantially longer life span [1,2,3]. Therefore, the use of microtechnology has increased over the last few years where micro-welding has gained significant importance for fabricating components with thin foils. It also demonstrates the high demand for various industrial applications including battery interconnect welding, micro-turbine fabrication and micro-electronics [4,5,6,7]. This micro-welding demands an efficient joining method which can address the joining challenges associated with ultra-thin foil welding, such as, overheating, distortion, warping and good part fit-up [8,9]. Therefore, attention is needed to precisely control heat input during the micro-welding process. One of such widely used micro-welding processes is micro-resistance spot welding (micro-RSW) which is a well-known joining technique for many industrial applications including thin foil joining due to low equipment cost, good quality control/process monitoring and low process variation [10,11]. However, micro-RSW is not free of challenges or issues. The main challenges associated with micro-RSW are surface cleaning of contaminants (i.e., grease, oil and dirt), RSW-electrode sticking with the parent materials, picking up the residue from the joint location and additional processes to maintain the original electrode shape (i.e., RSW electrode tip dressing) [12,13]. In addition, being a contact/touch-based joining process, micro-RSW is also a slow process, and obtaining a bigger nugget is difficult as it is limited by the RSW electrode size, parent material thickness and composition. In contrast, micro-laser spot welding offers several advantages over the micro-RSW process including non-contact joining, single-sided access, up to five times faster placement of spots, easy automation and flexibility in nugget size [14,15]. However, there are challenges associated with micro-laser welding as well, especially for joining ultra-thin sheets to thick sheets, such as (i) ensuring intimate contact between the mating surfaces [16], (ii) controlling heat input to avoid cutting through the thin upper sheet [17] and (iii) proper selection of laser welding parameters to control welding defects such as cracks, porosity or under-weld/no-weld conditions [18,19,20].

For micro-laser spot welding, especially of ultra-thin foils, pulsed laser welding is the preferred method of welding over continuous wave laser welding because it offers precise control of heat input reducing the heat-affected zone (HAZ), residual stresses and the presence of discontinuities [21]. Micro-laser welding (pulsed type) is used for joining many industrial components which are being made smaller and portable to save space, be lightweight and reduce energy consumption. Researchers have reported various micro-welding applications including medical device manufacturing, sensors with very thin membranes, welding of markers onto stents, fabrication of micro-turbine using ultra-thin foils, etc. [22,23,24,25]. One such micro-welding application is the joining of ultra-thin Inconel to stainless steel for micro-turbines where new challenges will appear that laser welding will have to address. In general, pulse laser welding is an intermittent supply of a high-intensity energy beam focused on material melting and solidification consecutively in a periodic manner. The process parameters in pulsed laser welding include pulse energy/laser power, pulse width, pulse frequency, laser spot size and welding speed which are used to obtain satisfactory weld quality [9,26]. Furthermore, for micro-laser spot welding, a spot can be created without moving the laser head, and welding speed might not be used during welding. To make the micro-laser spot, a few additional parameters can be considered such as wobble amplitude (to define the spot diameter if it is less than 1 mm), wobble frequency (rotation speed of the laser beam) and welding time (i.e., the duration used to turn on the laser beam).

Few researchers have reported the joining of Inconel with stainless steel using laser welding. For thicker material, Kim et al. [27] reported successful welding of Inconel 600 tubular components (stack-up of 1.0 mm to 1.2 mm Inconel) for nuclear power plants using a pulsed laser. Similar to this, investigations were conducted to laser-weld Inconel 625 (of 1.1 mm) and duplex stainless steel 2205 (DSS 2205 of 1.0 mm) in butt configuration [28], and narrower weld beads with improved mechanical properties were obtained with decreasing energy input. In another study by Ahmad et al. [29], the effect of process parameters on weld pool temperature distribution and mechanical properties were reported. Butt welding of 1.0 mm SS 304 and 1.0 mm Inconel 625 was investigated by Yan et al. [30]. Mechanical behaviours and microstructural properties of Inconel alloys as well as with steel dissimilar joining are reported in the literature [31,32,33]. However, these studies are in the category of thick sheet welding where the thickness of both sheets was above 1.0 mm. In the case of micro-laser welding, Ventrella et al. [21] investigated the effect of laser energy while welding AISI 316L stainless steel foil with 100 μm thickness, and they concluded that microstructural and mechanical reliability can be achieved by precisely controlling the laser pulse energy. In contrast, Lertora et al. [34] investigated CO_2_ laser overlap joints between Inconel 718 sheets of 0.4 mm to 1.6 mm, and they reported that despite microcracks, the fatigue behaviours of the overlap welds conformed to the requirements of Aircraft Engines. Lin et al. [35] studied the solidification hot cracking in laser-welded joints of Inconel 718 alloy foils of 0.2 mm. However, the direct comparison in terms of joint strength and microstructural properties using micro-resistance spot welding and micro-laser spot welding of Inconel foil to stainless steel sheet is missing.

This paper investigates the use of pulsed laser welding to produce micro-laser spot welds using a stack-up of 70 µm Inconel 718 ultra-thin foil to 1.0 mm thick 410 steel. The main objective of this research is to check the feasibility of producing high-quality and flexible laser joints to replace the existing micro-RSW process by evaluating the mechanical strength and microstructural characteristics of the welded specimens and, subsequently, joint performance characterisation at elevated temperature. Furthermore, reduction in joint strength at elevated temperature is to be compensated by increasing the number of laser spots such that the strength of the joint is at par with the ambient temperature test.

## 2. Experimental Setup and Procedure

**Figure 1:**
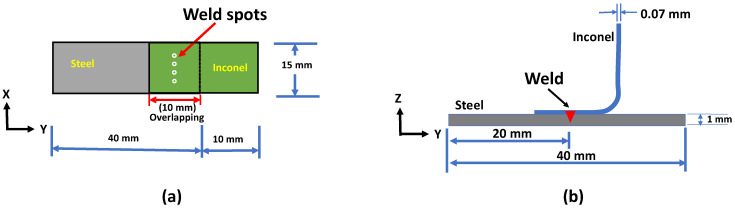
Schematic of tensile sample (**a**) lap shear specimen and (**b**) 90° peel specimen (sectional schematic view).

In this experimental work, the ultra-thin Inconel 718 foil of thickness 0.07 mm and 1.0 mm thick 410 steel of dimensions 20 mm × 15 mm and 40 mm × 15 mm were chosen for experimental investigations. The dimensions were chosen as per the guidelines received from the industrial advisor. The chemical compositions of workpieces are given in Table 1. As per the joining requirements, Inconel was used as the upper material and welded to steel in a lap joint configuration having four spots as shown in Figure 1, and a 0.5 mm diameter was used for each weld spot formation. In the joint configuration (Figure 1a), the overlap was chosen 5 mm on either side of the joint where the thickness of Inconel specimen was 0.07 mm. With less overlap, it was difficult to get sufficient space to place the joint as well as to ensure tight control of the part-to-part gap. Figure 1b shows a cross-sectional schematic image showing the location of the joint. Firstly, as per the existing joining method, micro-RSW was conducted using a MacGregor RSW machine having maximum output current flow of 6000 Amps DC with current control mode (make: MacGregor, Machida, Japan; model: M31 Smart Series). Dome-shaped RSW electrodes with 0.5 mm in diameter were used, and they were made of aluminum dispersed copper (Cu content around 98.9%). The micro-RSW welding parameters such as weld current (WC) and weld time (WT) were varied at 3 different levels because they had a significant effect on weld quality [11,36] whilst squeeze time (ST) and hold time (HT) were kept constant. After carrying out the initial screening tests (a few experimental trials using trial and error method), the process parameters and their limits (lower and upper limits) for the micro-RSW welding process are given in Table 2. In order to substitute the micro-RSW spots with laser spots, laser welding was conducted using a pulsed 1.5 kW YLR fibre laser (28 μm spot size) in peak power mode (make: IPG Photonics, Yokohama, Japan; model: Micro-Multi-axis Laser Processing Workcell) using clockwise circular wobbling [4,37] with a focal distance of 200 mm. An argon gas jet emerging from a nozzle coaxial with the laser beam at 20 L/min was used to avoid any external atmospheric contamination/oxidation during welding. The experiments were performed at different levels of laser power, pulse on time and pulse frequency whilst wobble frequency and wobble amplitude were kept fixed based on the results obtained from the initial screening. Four laser spots were produced as given in Figure 1a. The fixed process parameters and varied laser power, pulse on time and frequency values are tabulated in Table 2. A clamping device was used to maintain intimate contact between the two sheets. Statistical software Minitab v19 was applied to develop the response surface methodology (RSM) design matrix for systematic experiments for both micro-RSW and laser welding processes. Each experimental combination was replicated three times to obtain repeatable results. The welded samples for metallographic analysis were sectioned perpendicular to the welding direction. Samples for the metallographic analysis were prepared by polishing with successively finer SiC papers, down to 1200 grade, to remove the scratches. Each sample was then polished on 3 µm, 1 µm and 0.05 µm diamond solutions. Measurement of the 90° peel load of welded samples (Figure 1b) was conducted on an Instron 3367 test frame with a crosshead speed of 2 mm/min, and the weld bead geometry was analysed using an optical microscope (Nikon Eclipse LV150N, made: Nikon Metrology UK LTD, Derby, UK). Moreover, a JEOL 7800F field emission scanning electron microscope (FE SEM) equipped with an electron backscattered diffraction (EBSD) system was used for microstructural analysis.

## 3. Results and Discussions

The following section details the analysis of micro-RSW and laser-welded samples in terms of joint strength and metallography analyses. The welded joints were prepared under varying levels of process parameters as per Table 2.

### 3.1. Joint Strength Analysis

Joint strength is one of the most significant parameters to classify a weld as good or bad. A 90° peel load test was conducted to determine the weld/joint strength. The weld strength was defined in terms of the peak load which was obtained from the 90° peel load test. Each experiment was performed three times, and the average values of peak load (PL) were reported.

#### 3.1.1. Micro-RSW Joint Strength

**Figure 2:**
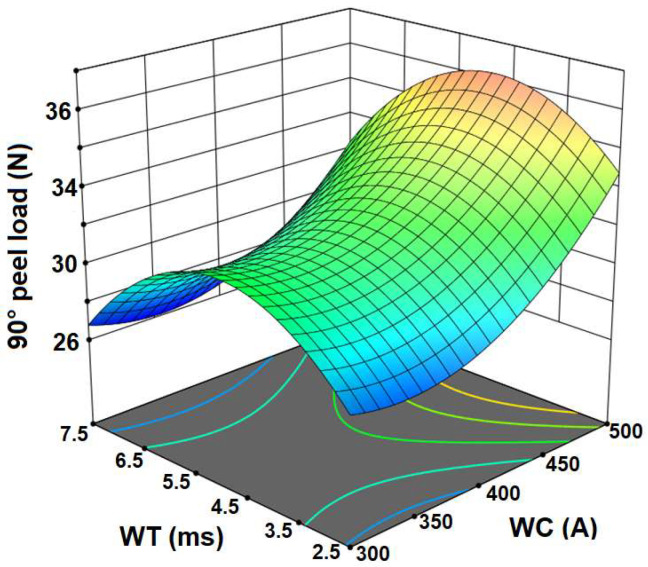
Response surface plots showing the interaction effects of weld current and weld time on the average peak load (PL).

The RSM design matrix and the measured values of average 90° peel peak load (PL) with standard deviation (SD) are given in Table 3. In Table 3, the process parameters were chosen as per central composite face-cantered design including four center points using RSM design (a second-order model can be developed efficiently) to analyze the response of interest based on the results. A second-order model can provide a better/efficient optimum value as well as interaction effect of process parameters compared to the linear model. The maximum and minimum PLs were obtained from sample no. 11 (i.e., 39.16 N) and sample no. 8 (i.e., 26.12 N), respectively. The maximum PL was obtained for the higher value of WC and the middle value of WT. Figure 2 shows 3D response surface and contour plots for the effect of process parameters on response which were constructed according to the fitted quadratic model. It is observed from Figure 2 that an increase in the weld time, up to some certain value, increases the PL. Further increase in the weld time reduced the PL observed. When the weld time was very low, incomplete penetration occurred. At longer weld times, excess melting of material was observed (burn-through of top sheet) with a consequent reduction in PL. Figure 2 also indicates that the PL increased with an increase in the value of weld current. Increasing the weld current increases the heat generation at the faying surface resulting in a large amount of the base metal being melted, consequently forming a weld with deeper penetration depth. It can also be concluded from Figure 2 that the maximum value of PL was obtained at the higher value of weld current and middle value of weld time. Similarly, the minimum value of PL was obtained within the range of lower and middle values of WC and the lower value of WT.

#### 3.1.2. Laser-Welded Joint Strength

**Figure 3:**
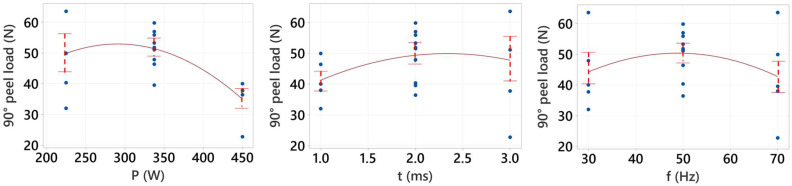
Main effects plot for means of 90° peel load.

The measured peak load (PL) responses obtained from the laser-welded 90° peel samples are listed in Table 4. In Table 4, the process parameters were chosen as per central composite face-cantered design including five center points using RSM design (a second-order model can be developed efficiently) to analyze the response of interest based on the results. A second-order model can provide a better/efficient optimum value as well as an interaction effect of process parameters compared to the linear model. The maximum and minimum PLs were observed for sample no. 20 (at P = 225 W, t = 3 ms and f = 30 Hz) and sample no. 14 (at P = 450 W, t = 3 ms and f = 70 Hz), respectively. Figure 3 shows the mean effect plot for the mean of 90° peel peak load which includes data point locations in blue color, mean plots and associated standard deviations. From Figure 3, it was found that the laser power has the most significant effect on the PL followed by pulse on time and pulse frequency. This is because the variation in the value of PL was maximum with the increase in laser power whilst minimum in the case of pulse frequency. It is apparent from the figure that PL increases with power and pulse frequency up to a threshold value and thereafter it starts decreasing. An initial increase in laser power and pulse frequency created a larger melt pool which elevated the quality of the weld from under-weld to good-weld; however, a further increase in laser power and pulse frequency created an undercut or underfill leading to an over-weld condition and subsequently reducing the PL [4,19,39]. Kumar et al. [37] achieved a similar trend of joint strength profile with laser power for the laser-welded 0.4 mm Al to 1.5 mm Al. In addition, PL increased continuously with the pulse on time; the underfilling of the top sheet material might not be reached within the limiting value of pulse on time. Consequently, the PL value is constantly increasing with the pulse on time.

**Figure 4:**
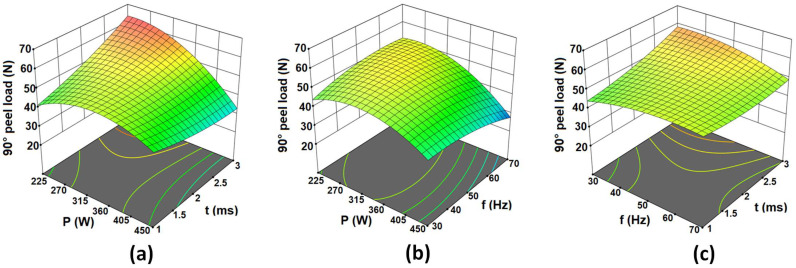
Response surface plots showing the interaction effects of (**a**) P and t, (**b**) P and f and (**c**) f and t on the PL, while the third parameter is at their respective centre value.

The 3D response surface and contour plots were constructed according to the fitted quadratic model as shown in Figure 4. Figure 4 shows the interaction plots for PL with one variable kept constant at their respective centre value and the other two within the working range. In terms of an interaction between the laser power and pulse on time, as shown in Figure 4a, the PL is maximum at lower values of laser power and higher values of pulse on time. This might be due to the lower power and higher pulse on time providing optimum weld profile between the sheets. It is evident from Figure 4b that PL increases with increasing pulse frequency at low power whilst decreasing at high laser power. This is because, at low power and low frequency, lower energy density is generated, resulting in a lack of penetration and thus a weak joint. Further, with a low value of laser power and pulse frequency towards the maximum value, the PL is improved as energy density increases and maximum strength is obtained. Meanwhile, at a high value of laser power and high pulse frequency, the depth of penetration is larger due to the higher rate of increase in specific point energy causing underfilling (overheating) and resulting in low joint strength [39]. From Figure 4c, it can be seen that by increasing the pulse frequency from 30 Hz to 70 Hz, PL increases for the low value of pulse on time whilst decreasing at high pulse on time. The maximum and minimum values of PL can also be observed from the 3D surface plots. It can be concluded from Figure 4a that the maximum values of PL can be achieved around the highest level of pulse on time (3 ms) and the lowest level of laser power (225 W). The exact values of the optimized process parameters to achieve maximum and reproducible PL are determined in Section 3.3. Maximum PL can therefore be achieved using the laser welding process (63.60 N) rather than the resistance spot welding process (39.16 N). As the main objective of this paper is to replace the existing micro-RSW process with flexible laser welding, optimization was conducted for the laser welding process.

### 3.2. Metallographic Analysis

To understand the morphology of the weld fusion zone from both welding processes, a metallographic analysis was conducted. This revealed key fusion zone characteristics for a better understanding of the welding behaviour.

#### 3.2.1. Micro-RSW Welding Process

**Figure 5:**
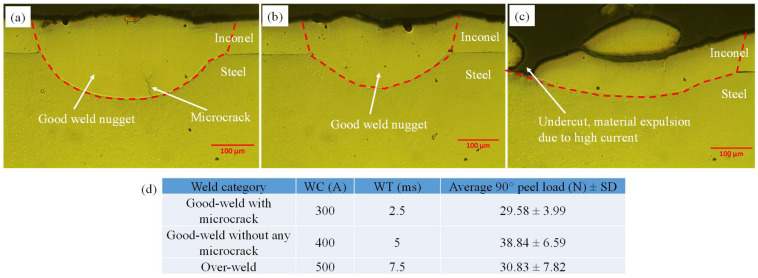
Optical micrographs of micro-RSW welded joints at (**a**) good weld with microcrack, (**b**) good weld without microcrack and (**c**) over-weld conditions with (**d**) the parameters used for the welding.

The weld interface of micro-RSW welds was studied with the help of optical micrographs, and it was found that within the selected input range of micro-RSW parameters (as indicated in Table 3), only two types of welds were observed, namely good welds and over-welds (Figure 5). Good welds could be categorised as good welds with a microcrack and good welds without a microcrack. A good weld with a microcrack appeared at WC = 300 A and WT = 2.5 ms (see Figure 5a). At WC = 400 A and WT = 5 ms, a good weld without a microcrack was observed (Figure 5b). It is evident from the micrographs that both the depth of penetration and interface width were high for the good weld. As a result, the T-peel load of the good weld without a microcrack was high (38.84 N) as indicated in Table 3. The appearance of the microcrack reduced the T-peel load to 29.58 N in good-weld joints as indicated in Table 3. The over-weld condition was prominent when WC = 500 A and WT = 7.5 ms were used. Undercut and material expulsion due to high current were the main attributes in micro-RSW over-weld joints (Figure 5c). As a result, the T-peel load was reduced (30.83 N) as the failure occurred at the location of the undercut in the over-weld.

**Figure 6:**
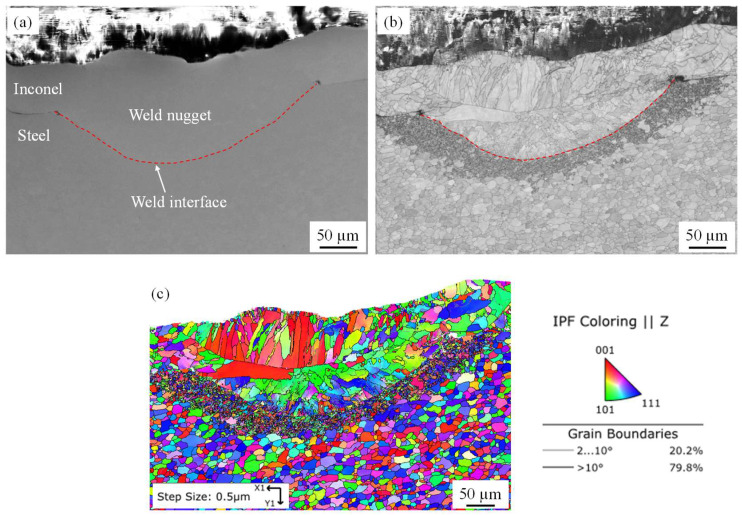
(**a**) SEM micrograph, (**b**) band contrast map and (**c**) EBSD map with IPF colouring of micro-RSW welded joint interface at good weld without any microcrack condition.

The weld microstructure of the micro-RSW welded joint was further studied by carrying out a SEM and EBSD analysis of the good weld without any microcracks condition. Figure 6a shows the SEM micrograph at the joint interface. There was no microcrack or void observed at the weld interface and at the weld zone. The band contrast image (Figure 6b) indicates that the interface is not prominent between the weld nugget on the Inconel side and steel base metal and there is a separate subgrain region between the weld nugget and steel base metal. The EBSD map shows that the average grain sizes of Inconel base metal, weld nugget, subgrain region and steel base metal are 10 µm, 18 µm, 2 µm and 9 µm, respectively (Figure 6c). The grains at the weld nugget were elongated from the weld interface to the middle of the weld nugget and again from the middle of the weld nugget to the top surface of the weld nugget. As there was a distinct subgrain region at the weld interface, the heat-affected zone in micro-RSW welding was large. The number of low-angle grain boundaries (LAGBs) as shown by the grey line (2° < grain boundary < 10°) is higher (20.2%) in micro-RSW good welds without any microcracks (Figure 6c). On the other hand, the number of high-angle grain boundaries (HAGBs) as shown by the black line (grain boundary > 10°) is comparatively lower (79.89%) in micro-RSW good welds without any microcracks (Figure 6c). As the HAGB provides greater hindrance to dislocation gliding, higher weld strength was achieved for the higher number of HAGBs [40].

#### 3.2.2. Laser Welding Process

**Figure 7:**
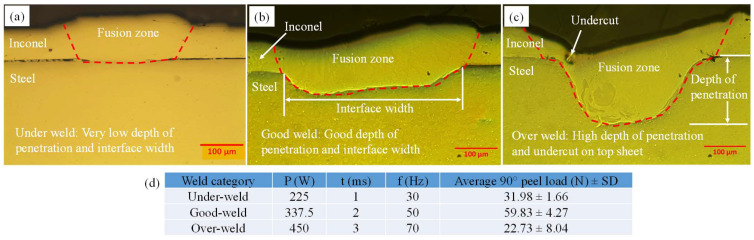
Optical micrographs of laser-welded joints in (**a**) under-weld, (**b**) good-weld and (**c**) over-weld conditions with (**d**) the parameters used for the welding.

The weld interface was studied with the help of optical micrographs to understand the joint morphology. In the selected input range of laser welding parameters as indicated in Table 4, three types of welds were observed, namely under-welds, good welds and over-welds (Figure 7). An under-weld appeared at P = 225 W, t = 1 ms and f = 30 Hz. It is shown in the under-weld micrograph that the depth of penetration and interface width were very low (Figure 7a) which reduced the T-peel load (31.98 N) as indicated in Table 4. At P = 337.5 W, t = 2 ms and f = 50 Hz, a good weld was observed. Good penetration depth and interface width were the main attributes of this type of joint (Figure 7b). As a result, the measured T-peel load in the good-weld condition was high (59.83 N) as shown in Table 4. An over-weld was observed at P = 450 W, t = 3 ms and f = 70 Hz. In the over-weld, the depth of penetration was high, but an undercut appeared on the top sheet (Figure 7c). Thus, the T-peel load was considerably reduced (22.73 N) as the failure happened at the location of the undercut in over-weld conditions.

**Figure 8:**
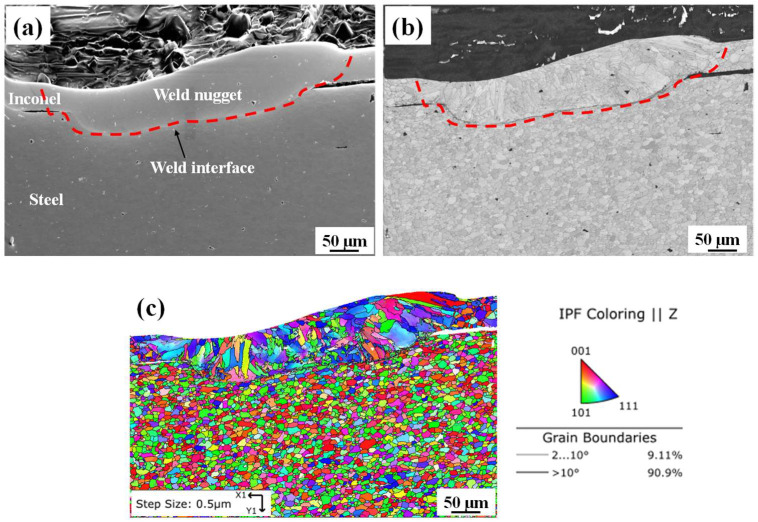
(**a**) SEM micrograph, (**b**) band contrast map and (**c**) EBSD map with IPF colouring of laser-welded joint interface at good-weld condition.

The weld microstructure of the laser-welded joint was further studied using the scanning electron microscope (SEM) and electron backscatter diffraction (EBSD) analysis for good-weld conditions. Figure 8a shows the SEM micrograph of the fusion zone. There was no microcrack or void observed at the weld interface or at the weld zone. The band contrast image (Figure 8b) shows a prominent interface between the weld nugget in the Inconel and the steel base metal. There was no subgrain region in between the weld nugget and steel base metal. The EBSD map shows that the average grain sizes of Inconel base metal, weld nugget and steel base metal are 10 µm, 23 µm and 9 µm, respectively (Figure 8c). The grains at the weld nugget were elongated from the weld interface to the top surface of the weld nugget. As there was no subgrain region at the weld interface, the heat-affected zone in laser welding was much smaller than observed in micro-RSW. The number of low-angle grain boundaries (LAGBs) as shown by the grey line (2° < grain boundary > 10°) is lower (9.11%) in the laser-welded good weld (Figure 8c). The number of high-angle grain boundaries (HAGBs) as shown by the black line (grain boundary > 10°) is higher (90.89%) in the laser-welded good weld (Figure 8c). As the HAGB provides greater hindrance to dislocation gliding, higher weld strength was achieved [40]. Thus, the laser-welded joints prepared under the good-weld condition provide better weld strength than the corresponding micro-RSW welds.

### 3.3. Detailed Analysis of Laser Welding Process Parameters

The surrogate models for response (Equation (1)), which can be used for prediction within the same design space, in terms of actual factors are shown below:

(1) $$\begin{array}{cc} 90{}^{\circ}\;peel\;load\;\left(N\right) & =-116.1+0.617\times P+28.5\times t+1.75\times f-0.0698\times P\times t-0.001942\times P\times f\\ & -0.193\times t\times f-0.000662\times P^{2}+1.98\times t^{2}-0.00750\times f^{2} \end{array}$$

The adequacy of the developed model and the test of significance of model coefficients were performed using the sequential analysis-of-variance (ANOVA) technique using the Minitab v19 software to obtain the best-fit models. The associated *p*-value of less than 0.05 for the model (i.e., *p*-value < 0.05, at 95% confidence level) indicates that the model terms are statistically significant. The ANOVA results (Table 5) also show that the other adequacy measure, i.e., *R*^2^, for the response was greater than 80%, which is reasonable and indicates the model is effective [19,39]. The ANOVA indicates that (Table 5) the laser power (*P*), the interaction effect of laser power and pulse on time (*P* × *t*) and the quadratic effect of the laser power (*P*^2^) are the significant model terms. Other model terms were statistically insignificant.

**Figure 9:**
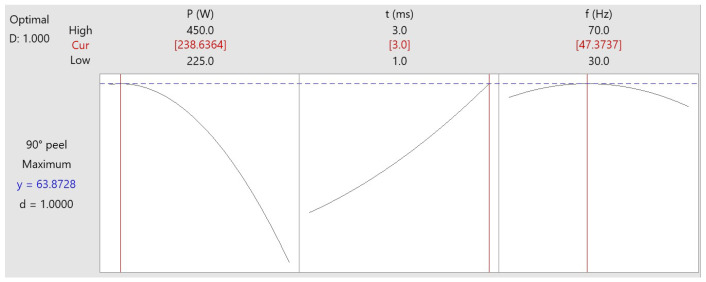
Optimization results for average maximum peak load (PL).

Desirability function analysis is one of the most widely used approaches in the industry for the optimization of response [19,37,38]. Single-objective optimization for laser welding of Inconel to steel was carried out, and the optimized predicted results of average PL are shown in Figure 9. The goal was to maximize PL, which is desired for good-quality welds. To obtain the desired PL value, equal importance was given to the upper and lower bounds of the process parameters and the target value of the linear desirability function. For the linear desirability function (d), the value of the weight is considered to be 1 (for the response, a desirability function assigns numbers between 0 and 1; 0 represents a completely unwanted value, and 1 represents a completely appropriate or ideal PL value). In Figure 9, each row of the graph corresponds to a response variable (Equation (1)) and each column corresponds to one of the process parameters. Each cell of the graph shows how one of the response variables changes as a function of the process parameters, keeping other parameters constant. The vertical line inside the graph indicates the optimum parameter combination, and the horizontal dotted line represents the optimised response values.

The numbers displayed at the top of the column show the upper and lower limits of laser welding parameters with the optimum parameter level setting in red colour. On the left side of the row, the goal set for the PL for the optimization, the projected PL (y) value at the optimum parametric combination and the individual desirability value (=1) are given. The optimum value of PL (63.87 N) was obtained at a laser power of 238.63 W, pulse on time of 3 ms and pulse frequency of 47.37 Hz. The value of the composite desirability factor (D) was 1 (i.e., the individual desirability was combined using the geometric mean, which gave the overall/composite desirability “D”).

#### 3.3.1. Confirmatory Tests

The predicted result of the PL obtained by desirability function analysis was validated by conducting confirmatory tests. Three confirmatory experiments were conducted at the optimum parametric combination. The results of experiments at optimum conditions are given in Table 6. It can be seen from the table that there is less than 3% between the predicted/optimum and the experimental values, which validates the applied optimization technique.

#### 3.3.2. Ambient vs. Elevated Temperature Tests

**Figure 10:**
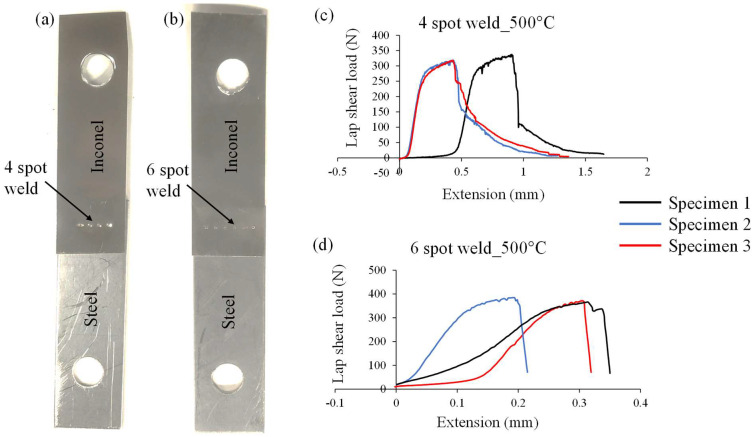
Laser weld with (**a**) 4 spots and (**b**) 6 spots. Load vs. extension plot of lap shear test at 500 °C temperature for (**c**) 4-spot and (**d**) 6-spot laser welds.

As these joints will be operating at elevated temperatures during their functional use, ambient vs. elevated temperature characterisation is also important to understand the behaviour of the joints. The lap shear tests (three repetitions) were conducted for the laser-welded Inconel to steel samples, prepared at optimized parameter settings determined using desirability function analysis presented in Section 3.3 (Figure 9), at room temperature (i.e., ~23 °C) and 500 °C. The results are shown in Table 7. The average lap shear load at the elevated temperature (324.59 N) was about 17% less than the lap shear load at ambient temperature (390.31 N). To achieve an average shear load at elevated temperature (500 °C) nearly equal to that at ambient temperature, the number of weld spots was increased by 2 (i.e., six spots) as shown in Figure 10. The results are shown in Table 8. The average lap shear load obtained at elevated temperature for six spots (374.15 N) was increased by about 16% over the four-spot weld (324.59 N) and was about 4% lower than the average lap shear obtained at ambient temperature (390.31 N). Being a flexible joining process for placing the spots and a faster joining process, laser welding exhibited added advantages over the micro-RSW process.

## 4. Conclusions

This paper presents a comparative study between laser and micro-RSW welding of Inconel to stainless steel joints by conducting mechanical and metallurgical characterization. The results suggest that the laser welding process is a suitable welding process to achieve high-quality welded joints between parts or components comprising thin Inconel foils which are exposed to elevated temperature environments. From the foregoing analysis and discussion, the following conclusions were drawn:

➢ From mean effect plots, it was found that for micro-RSW, the weld current had the most significant effect on the 90° peel load followed by weld time. Similarly, in the case of laser welding, laser power had a significant effect on the 90° peel load followed by pulse on time and pulse frequency within the selected working range of the process parameters.
➢ The micro-RSW welded joints showed relatively lower weld strength than laser-welded joints under good-weld parametric conditions.
➢ This paper identified the optimized process parameters for laser welding processes to achieve good weld for thin Inconel to steel joints.
➢ The laser weld provided maximum joint strength of about 63.60 N which was significantly higher than the micro-RSW welding process of about 39.16 N.
➢ For four spots of laser weld, the average lap shear load obtained for the laser welding of Inconel to steel joints at elevated temperature (324.59 N) was lower (about 17%) than the ambient temperature (390.31 N). Meanwhile, for six spots of laser weld, the average lap shear load (374.15 N) was about 4% lower than the average load at ambient temperature.
➢ In further work, a detailed study may be conducted to establish the relationship of weld quality with laser seam shape instead of spot.

## Figures and Tables

**Table 1:** Chemical composition (wt%) of work materials similar materials are used in [33,38].

Materials	Thickness (mm)	Chemical Compositions (wt%)
IN 718	0.07	Ni = 52.1, Cr = 17.69, Nb = 4.9, Mo = 2.9, C = 0.031, Fe = 20.7, Cu = 0.11, P = 0.09, Al = 0.29, Ti = 0.72, Co = 0.36, S = 0.012, Si = 0.1 and Mn = 0.2
410 steel	1.0	C = 0.15%, Mn = 1.00%, Si = 1.00%, Cr = 11.5–13.5%, P = 0.04%, S = 0.03% and Fe balance

**Table 2:** Process parameters and their limits.

Type of Joints	Varied Parameters	Fixed Parameters	Levels		
			1	2	3
Micro-RSW	Weld current (WC), A	ST = 50 ms; HT = 100 ms	300	400	500
	Weld time (WT), ms		2.5	5	7.5
Laser weld	Laser power (P), W	Wobble amplitude = 0.5 mm Wobble frequency = 600 Hz	225	337.5	450
	Pulse on time (t), ms		1	2	3
	Pulse frequency (f), Hz		30	50	70

**Table 3:** Experimental design and measured response.

Experiment No.	Process Parameters		Response
	WC (A)	WT (ms)	Average 90° Peel Load (N) ± SD
1	400	7.5	29.00 ± 6.64
2	400	5	30.20 ± 10.32
3	500	2.5	35.52 ± 7.46
4	400	5	27.25 ± 1.02
5	300	5	30.84 ± 9.75
6	300	2.5	29.58 ± 3.99
7	500	7.5	30.83 ± 7.82
**8**	**400**	**2.5**	**26.12** ± 6.33
9	400	5	38.84 ± 6.59
10	400	5	32.98 ± 4.59
**11**	**500**	**5**	**39.16** ± 9.35
12	300	7.5	26.19 ± 7.36

**Table 4:** Experimental design and measured response.

Experiment No.	Process Parameters			Response
	P (W)	t (ms)	f (Hz)	Average 90° Peel Load (N) ± SD
1	337.5	1	50	46.37 ± 0.54
2	337.5	2	50	59.83 ± 4.27
3	450	1	30	40.03 ± 2.77
4	337.5	2	50	53.28 ± 7.26
5	337.5	2	50	51.79 ± 4.69
6	225	1	30	31.98 ± 1.66
7	337.5	2	70	39.58 ± 0.89
8	337.5	2	50	55.88 ± 2.90
9	337.5	2	50	56.96 ± 3.62
10	337.5	3	50	51.07 ± 1.02
11	450	2	50	36.41 ± 6.67
12	225	1	70	49.90 ± 6.74
13	337.5	2	30	47.90 ± 4.89
**14**	**450**	**3**	**70**	**22.73** ± 8.04
15	225	3	70	63.58 ± 2.69
16	450	3	30	37.72 ± 5.24
17	450	1	70	37.97 ± 2.36
18	225	2	50	40.31 ± 3.59
19	337.5	2	50	51.48 ± 1.69
**20**	**225**	**3**	**30**	**63.60** ± 4.24

**Table 5:** ANOVA for the fitted quadratic model for response.

ANOVA Terms	Response		
	90° Peel Load (N)		
Source	*F*-Value	*p*-Value	Remarks
Model	5.42	0.007	Significant
*P*	14.55	0.003	Significant
*t*	2.76	0.128	Non-significant
*f*	0.15	0.710	Non-significant
P×t	12.94	0.005	Significant
P×f	4.00	0.073	Non-significant
t×f	3.12	0.108	Non-significant
*P* ^2^	5.06	0.048	Significant
*t* ^2^	0.28	0.607	Non-significant
*f* ^2^	0.65	0.439	Non-significant
*R* ^2^	83%		

**Table 6:** Confirmatory/validation test results.

The Optimum Condition for PL (N)			Confirmatory Experimental Results			|Error%|
Process Parameters	Values	PL (N)	Trials	PL (N)	Average ± SD	
*P* (W)	238.63	63.87	Test 1	66.39	65.4 ± 0.91	2.4
*t* (ms)	3		Test 2	65.23		
*f* (Hz)	47.37		Test 3	64.58		

**Table 7:** Lap shear test at normal and elevated temperature.

Test Condition	Lap Shear Force (N)				
	Test 1	Test 2	Test 3	Average PL ± SD	% Change From Ambient to Elevated Temperature
Ambient temperature (23 °C)	390.18	389.13	391.63	390.31 ± 1.25	16.84
Elevated temperature (500 °C)	337.44	317.98	318.36	324.59 ± 11.24	

**Table 8:** Lap shear test of welds with 6 spots at elevated temperature.

Test Condition	Lap Shear Load (N)			
	Sample 1	Sample 2	Sample 3	Average Load ± SD
Elevated temperature (500 °C)	371.82	384.60	366.04	374.15 ± 9.50

## Data Availability

Not applicable.
